# Supplementary material for: Using social marketing for the promotion of cognitive health: a scoping review protocol
Source: BMJ Open. 2021 Oct 13;11(10):e049947. doi: 10.1136/bmjopen-2021-049947 (PMC8515474; doi:10.1136/bmjopen-2021-049947)
Supplement: Supplementary data [file bmjopen-2021-049947supp001.pdf]

Data extraction instrument

A. General Information

1. **Date form completed** *(dd/mm/yyyy)*	
2. **Name/ID of person extracting data**	
3. **Report title** *(title of paper/ abstract/ report that data are extracted from)*	
4. **Report ID** *(if there are multiple reports of this study)*	
5. **Reference details**	
6. **Report author contact details**	
7. **Publication type** *(e.g. full report, abstract, letter)*	
8. **Study funding source** *(including role of funders)*	
9. **Possible conflicts of interest** *(for study authors)*	
10. **Notes:**	

B. Eligibility

**Study Characteristics**	**Review Inclusion Criteria** *(Consider inclusion criteria for each characteristic as defined in the Protocol)*	**Yes/ No / Unclear**	**Location in text** *(pg/fig/table)*
11. **Type of study**	Design (specify):	...	
12. **Participants**		...	
13. **Types of intervention**		...	

**Study Characteristics**	**Review Inclusion Criteria**<br>*(Consider inclusion criteria for each characteristic as defined in the Protocol)*	**Yes/ No / Unclear**	**Location in text**<br>*(pg/fig/table)*
14. **Types of outcome measures**		...	
15. **Decision:**	...		
16. **Reason for exclusion**			
17. **Notes:**			

DO NOT PROCEED IF STUDY EXCLUDED FROM REVIEW

C. *Population and setting*

	**Description** *(Include comparative information for each group (i.e. intervention and controls) if applicable)*	**Location in text** *(pg/fig/table)*
18. **Population description** *(from which study participants are drawn)*		
19. **Setting** *(including location and social context)*		
20. **Inclusion criteria**		
21. **Exclusion criteria**		
22. **Method(s) of recruitment of participants**		
23. **Notes:**		

D. *Methods*

	**Descriptions as stated in report/paper**	**Location in text** *(pg/fig/table)*
24. **Aim of study**		
25. **Design** *(e.g. RCT, non-RCT, etc.)*		
26. **Unit of allocation** *(by individuals, cluster/ groups or body parts)*		
27. **Start date**		
28. **End date**		
29. **Duration of participation** *(from recruitment to last follow-up)*		

30. **Notes:**

E. Risk of Bias assessment

**Domain**[1]	**Risk of bias** *Low/ High/Unclear*	**Support for judgement**	**Location in text** *(pg/fig/table)*
31.	…		
32.	…		
33.	…		
34.	…		
(expend rows as needed)	…		
35. **Notes:**			

F. Participants

	**Description as stated in report/paper**	**Location in text** (pg/fig/table)
36. **Total pop. at start of study**		
37. **Withdrawals and exclusions**		
38. **Total participants included in the study**		
(For the following sections, from 39 to 43, consider only the participants included)		
39. **Age**		

[1] *For randomised studies, see Chapter 23.1.2 of the Cochrane Handbook:* *https://training.cochrane.org/handbook/current/chapter-23#_Ref529610397*

For non-randomised studies, see Chapter 24.5 of the Cochrane Handbook: *https://training.cochrane.org/handbook/current/chapter-24#section-24-5*

	**Description as stated in report/paper**	**Location in text**<br><br>(pg/fig/table)
40. **Sex**		
41. **Race/Ethnicity**		
42. **Other relevant sociodemographics**		
43. **Other relevant characteristics in relation to the study/intervention** *(e.g. knowledge of a disease)*		
44. **Notes:**		

G. Procedure

In case of multiple groups, copy and paste the table for each intervention and comparison group, and title tables as "intervention group 1", "intervention group 2", etc.

	**Description as stated in report/paper**	**Location in text**<br><br>(pg/fig/table)
45. **Group name (if applicable)**		
46. **No. randomised to group (if applicable)**		
47. **Description** *(include sufficient detail for replication, e.g. content, components, measures, delivery; if applicable, describe the pre-intervention)*		
48. **Duration**		
49. **Timing (if applicable)** *(e.g. duration of each stage)*		
50. **Providers** *(e.g. no., profession, training, ethnicity etc. if relevant)*		

	**Description as stated in report/paper**	**Location in text**<br><br>(pg/fig/table)
51. **Co-interventions (if applicable)**		
52. **Economic variables** *(i.e. intervention cost, changes in other costs as result of intervention)*		
53. **Resource requirements to replicate intervention** *(e.g. staff numbers, equipment…)*		
54. **Notes:**		

H. Results

If applicable, duplicate the table to present results details for each outcome

	**Description as stated in report/paper**	**Location in text** *(pg/fig/table)*
55. **Outcome**		
56. **Subgroup (if applicable)**		
57. **Time point** *(specify whether from start or end of intervention)*		
58. **Results** *(Note changes from baseline data and provide any statistical relevant details (e.g., means, standard deviations, confidence intervals, exact p-values); at least, refer to the appropriate in-text table or figure)*		
59. **Mediating variables**		
60. **Moderating variables (and intermediate results)**		

	**Description as stated in report/paper**	**Location in text** *(pg/fig/table)*
61. **No. missing participants and reasons**		
62. **Unit of analysis** *(e.g. by individuals, health professional, practice, hospital, community; was the unit of analysis independent from the provider?)*		
63. **Statistical methods used and appropriateness of these methods** *(e.g. adjustment for correlation)*		
64. **Notes:**		

I. Applicability

	**Yes/No/Unclear**	**Notes:**
65. **Have important populations been excluded from the study?** *(consider disadvantaged populations, and possible differences in the intervention effect)*	...	
66. **Is the intervention likely to be aimed at disadvantaged groups?** *(e.g. lower socioeconomic groups)*	...	
67. **Does the study directly address the review question?** *(any issues of partial or indirect applicability)*	...	
68. **Notes:**		

J. Other information

	**Description as stated in report/paper**	**Location in text** *(pg/fig/table)*
69. **Key conclusions of study authors**		
70. **References to other relevant studies**		
71. **Further study information requested** *(from whom, what and when)*		
72. **Correspondence received** *(from whom, what and when)*		
73. **Notes:**		
